# Oral Supplementation of *Lasia spinosa* Thwaites Improves Sperm Cryotolerance Without Markedly Affecting Hematological, Biochemical, Seminal, or Testicular Profiles in Dogs

**DOI:** 10.3390/ani15162379

**Published:** 2025-08-13

**Authors:** Thitiporn Thongsima, Thitida Pakdeesanaeha, Sirichai Techarungchaikul, Ratree Jintana, Norraset Towanabutr, Sawita Santiviparat, Sudchaya Bhanpattanakul, Larindhorn Udomthanaisit, Theerawat Tharasanit

**Affiliations:** 1 Obstetrics, Gynaecology and Reproduction Unit, Small Animal Teaching Hospital, Faculty of Veterinary Science, Chulalongkorn University, Bangkok 10330, Thailand; tm.thongsima@gmail.com (T.T.); toey.duckool@gmail.com (T.P.); sirichai.t@chula.ac.th (S.T.); 2 The College of Veterinary Specialties of Thailand, Nonthaburi 11000, Thailand; 3 Department of Obstetrics, Gynaecology and Reproduction, Faculty of Veterinary Science, Chulalongkorn University, Bangkok 10330, Thailand; larin.udtns@gmail.com; 4 The Research and Development Center for Livestock Production Technology, Faculty of Veterinary Science, Chulalongkorn University, Bangkok 10330, Thailand; ratree.j@chula.ac.th; 5 Veterinary Medicine and Animal Husbandry Sub-Division, Patrol and Special Operations Division, Metropolitan Police Bureau, Bangkok 10210, Thailand; iamyoosg@hotmail.com; 6 Center of Excellence for Veterinary Clinical Stem Cells and Bioengineering, Chulalongkorn University, Bangkok 10330, Thailand; sawita.s@chula.ac.th (S.S.); sudchaya73@gmail.com (S.B.); 7 Department of Surgery, Faculty of Veterinary Science, Chulalongkorn University, Bangkok 10330, Thailand; 8 Department of Veterinary Technology, Faculty of Veterinary Technology, Kasetsart University, Bangkok 10900, Thailand

**Keywords:** canine, cryopreservation, frozen sperm, *Lasia spinosa* Thwaites (LST), semen quality, testosterone

## Abstract

*Lasia spinosa* Thwaites (LST) has emerged as a potential supplement for enhancing male reproductive performance. This study evaluated the effects of long-term oral supplementation with LST on hematological parameters, semen characteristics, ultrasonographic measurements of the prostate gland and testes, and the cryopreservation potential of canine sperm. Six healthy male dogs received oral LST supplementation at a dosage of 10 mg/kg body weight once daily for 7 days (short-term). After a three-month washout period to ensure full physiological recovery, the same dogs underwent a long-term supplementation protocol (60 days). In the short-term trial, no clinically significant changes were observed in hematological or serum biochemical parameters, including complete blood count, alanine aminotransferase, creatinine, blood urea nitrogen, total protein, and albumin; all parameters were within normal reference ranges. Serum testosterone levels and semen characteristics were also unaffected (*p* > 0.05). During the long-term treatment, blood profiles and testosterone levels remained stable. Although prostatic and testicular volumes increased slightly, the changes were not statistically significant (*p* > 0.05). A significant increase in semen volume was observed (*p* < 0.05), while other semen parameters showed no significant differences. Notably, post-thaw sperm motility significantly improved at both 15 min and 4 h after thawing, and sperm viability was significantly enhanced at 4 h post-thaw (*p* < 0.05), suggesting a potential protective effect of LST during cryopreservation. These findings indicate that LST supplementation is physiologically safe and may improve canine sperm quality during freezing and thawing, supporting its potential application in reproductive health management.

## Simple Summary

*Lasia spinosa* Thwaites (LST) is a natural herbal supplement that did not produce any adverse changes in blood test results following either short- or long-term administration in male dogs. Although testosterone levels and fresh semen quality were not significantly affected, long-term LST supplementation improved the post-thaw quality of sperm. These findings suggest that LST may be a promising option for supporting sperm cryopreservation in dogs.

## 1. Introduction

Managing canine reproduction is a fundamental aspect of veterinary practice, particularly within breeding programs. The quality of canine sperm used for artificial insemination is a critical factor influencing fertility rates. This is closely linked to spermatogenesis—a highly regulated biological process involving successive mitotic and meiotic divisions of spermatogenic cells [1,2]. These cellular processes lead to the production of mature, functional spermatozoa capable of successful fertilization. Sperm quality is assessed based on several parameters, including motility, viability, morphology, and total sperm per ejaculate, all of which can be affected by factors such as environmental stress, age, and nutritional status [3]. Moreover, sperm quality significantly influences conception rates, particularly in artificial insemination, for which both fresh and frozen–thawed semen are commonly used [4]. The use of frozen–thawed semen in artificial insemination presents challenges because cryopreservation can impair the sperm’s capacity for capacitation and the acrosome reaction—both of which are essential for fertilization [5]. As a result, frozen–thawed semen often exhibits reduced motility and viability due to irreversible cryodamage incurred during the freezing and thawing process. Although the exact mechanisms are not fully understood, the damage is largely attributed to the excessive formation of ice crystals and oxidative stress affecting cellular structures such as sperm membranes and mitochondria [6]. To enhance semen quality prior to use, various nutritional supplements have been employed. These include vitamins, minerals, antioxidants, and herbal extracts, all of which play crucial roles in sperm metabolism, viability, and function [7,8,9]. Despite some positive effects on reproductive health, many of these supplements have shown limited efficacy. Furthermore, prolonged use has been associated with potential adverse effects, including hormonal imbalances and toxicity. Long-term use of certain supplements may compromise liver and kidney function due to metabolic overload and the body’s inability to effectively process excessive doses [10,11,12].

The herbal supplement *Tribulus terrestris*, a medicinal plant, has been shown to enhance libido and improve the quality of chilled sperm in dogs [13], while vitamin E serves as a powerful antioxidant that protects sperm cells from oxidative stress and promotes fertility. However, the long-term effects of this herb on overall health and the freezing ability of canine sperm remain unclear. *Lasia spinosa* Thwaites (LST), a plant native to Southeast Asia, has emerged as a promising supplement for enhancing male reproductive performance because of its high content of phytoandrogens [14], which stimulate androgenic activity in various species. Specifically, LST administration has been linked to improvements in sperm quality in boars [15], rooster [16], and buffalo [17], underscoring its potential as a species-transcending supplement for reproductive support.

Phytochemical analyses suggest that compounds such as flavonoids, steroids, and saponins in LST contribute to its positive effects on semen quality and testosterone production. In rams, LST administration has been shown to increase circulating testosterone levels and improve sperm concentration and morphology [18]. These reproductive benefits are believed to result from the combined actions of phytoandrogens and antioxidant compounds in LST, including phenolic acids such as syringic acid, morin, and apigenin [19]. By mitigating oxidative stress and preserving sperm membrane integrity, these bioactive constituents provide a strong rationale for evaluating serum testosterone levels and the potential cryoprotective effects of LST in the present study. However, the use of LST as a dietary supplement for reproductive health in dogs remains largely unexplored. This study aimed to evaluate the short- and long-term effects of oral LST supplementation on hematological and biochemical parameters in dogs, including complete blood count (CBC), alanine aminotransferase (ALT), alkaline phosphatase (ALP), blood urea nitrogen (BUN), creatinine, total protein (TP), albumin, serum testosterone levels, and semen quality. In addition, the long-term effects on testicular and prostate volumes, as well as sperm cryopreservation ability, were examined.

## 2. Materials and Methods

### 2.1. Animals

The use of animals and the research methodology in this study were approved by the Chulalongkorn University Animal Care and Use Committee (IACUC), Chulalongkorn University (Animal Use Protocol No. 2231046). All procedures were conducted in accordance with relevant guidelines and regulations and adhered to the ARRIVE guidelines. Six healthy male dogs, aged 2–3 years (five Labrador retrievers and one German shepherd), were selected from the Police K-9 Division in Bangkok, Thailand, where they were permanently housed throughout the entire study period. All dogs were singly housed in semi-indoor enclosures under routine management conditions, and no relocation or housing changes occurred during the study. None of the animals had previously undergone semen collection or been exposed to pharmacological agents or chemical substances. The dogs had ad libitum access to fresh water and were fed a commercial dry food (LuvCare™ Active, Adult Large Breed; Bangkok, Thailand), which they had been accustomed to prior to study initiation.

### 2.2. Lasia Extraction and Biochemical Assay

Whole plants of LST were collected from Suphan Buri Province, Thailand (14.5379° N, 99.9912° E), located in the central region of the country. Species authentication was performed by comparison with herbarium specimens deposited at the Department of Botany, Faculty of Science, Chulalongkorn University, Thailand.

The rhizomes and roots of LST were collected and processed into LST powder following a method modified from Suthikrai et al [17]. Briefly, the underground parts of fresh LST plants were manually sliced to a thickness of approximately 2–3 mm and dried at 40 °C in a hot air oven until a moisture content of 10–13% was achieved. The dried material was ground using a mechanical grinder and sieved to obtain a uniform fine powder, which was then encapsulated and administered orally at an individual dose of 10 mg/kg body weight per day. The moist controlled LST powder was analyzed for testosterone concentration and antioxidant activity, which was determined using a radioimmunoassay (RIA) and enzyme-linked immunosorbent assay (ELISA), respectively. Testosterone levels in different batches ranged from 0.19 to 0.92 ng/g dry weight [17]. For the present study, the LST powder used was confirmed to contain a testosterone concentration of 0.4 ng/g dry weight.

The antioxidant potential of the sample was assessed using the DPPH Antioxidant Assay Kit (Dojindo Molecular Technologies, Kumamoto, Japan), following the manufacturer’s protocol. Absorbance was measured at 517 nm after a 30 min incubation in the dark to evaluate the reduction in DPPH radicals. Trolox, a well-established antioxidant standard, was used as the reference compound. The antioxidant capacity of the sample was expressed as the Trolox Equivalent Antioxidant Capacity (TEAC), calculated from the IC_50_ values of both Trolox and the extract. The LST extract showed a concentration-dependent scavenging effect against DPPH radicals. Its IC_50_ was determined to be 2.76 µg/mL, while that of Trolox was 5 µg/mL. Accordingly, the TEAC value of the extract was calculated to be 1.81, indicating strong antioxidant potential.

### 2.3. Experimental Design

**Figure 1:**
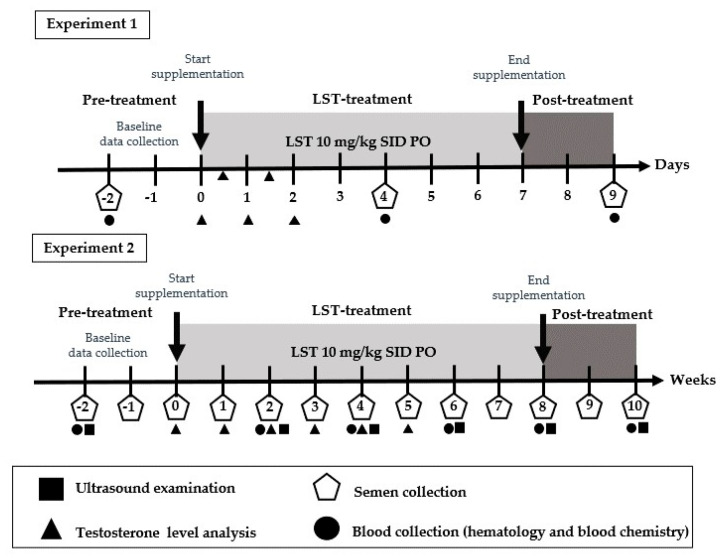
Experimental design and sampling timeline for Experiment 1 (short-term) and Experiment 2 (long-term) LST supplementation. Experiment 1 (short-term): Baseline data were collected on Day 2 for hematological values and semen quality. Serum testosterone was measured on Day 0 prior to LST administration. LST was administered orally at a dose of 10 mg/kg once daily from Day 0 to Day 6 (gray area). After the first dose, serum testosterone was assessed four times during the treatment period at 12 h intervals. Interim evaluations of hematological values and semen quality were conducted on Day 4, and post-treatment assessments were performed on Day 9, two days after the final administration. Experiment 2 (long-term): Baseline evaluations were conducted at Week 2 for hematological values and ultrasonographic measurements of the prostate gland and testes. Semen samples were collected at Week 2 and Week 1 to establish baseline quality. Baseline serum testosterone was measured at Week 0 prior to LST administration. LST was administered orally at a dose of 10 mg/kg daily for 60 consecutive days (gray area). During the treatment period, hematological values were assessed biweekly until Week 10 (two weeks post-supplementation). Serum testosterone levels were measured weekly from Week 1 to Week 5. Semen samples were collected weekly from the initiation of LST supplementation to Week 10.

In this study, dogs received orally administered LST extract at a dose of 10 mg/kg once daily in the morning before feeding. The study was structured into two distinct experiments, as illustrated in Figure 1.

Experiment 1 focused on evaluating the short-term effects of LST supplementation on hematological parameters. Dogs were administered LST orally for 7 consecutive days. Blood samples were collected at three time points: two days prior to supplementation (baseline), on Day 4 of treatment, and two days after the final dose to evaluate immediate physiological responses and recovery. Semen characteristics were assessed concurrently with each blood collection. Additionally, serum samples for testosterone level analysis were obtained twice daily at 7:00 a.m. and 7:00 p.m.

Experiment 2 involved the daily administration of LST (10 mg/kg) each morning for 60 consecutive days, corresponding to the duration of one full spermatogenic cycle in dogs. From the start of treatment, blood sampling and ultrasonographic evaluations of the testes and prostate were conducted twice weekly. Semen samples and serum testosterone levels were collected weekly to monitor reproductive function and hormonal dynamics. Semen quality was assessed in both fresh and cryopreserved–thawed samples to evaluate the impact of long-term LST supplementation on sperm freezability.

### 2.4. Ultrasonography for Testicular Evaluation and Prostate Gland Volume

Ultrasonography was performed using a Mindray M9 Portable Ultrasound equipped with a 5 to 8 MHz micro-convex probe (Shenzhen Mindray Bio-Medical Electronics, Shenzhen, China). Dogs were positioned in dorsal recumbency, and acoustic gel was applied. The transducer was initially placed on the lateral surface of the testis, capturing longitudinal and transverse B-mode images, with the mediastinum serving as a reference for testicular dimensions. Testicular volume was calculated using the ellipsoid formula [20]:

$$Volume\left({cm}^{3}\right)=length\times width\times height\times 0.71$$

Prostate gland volume was measured from longitudinal views, using the hypoechoic urethral tract as a reference. Prostate volume was estimated using the following formula [21]:

$$Volume\left({cm}^{3}\right)=\left(\frac{1}{2.6}\times length\times width\times height\right)+1.8$$

All ultrasonographic examinations were conducted in the morning, immediately after blood sample collection, to ensure consistent physiological conditions. Scans were performed only during Experiment 2—specifically at Week 2 (pre-treatment), every two weeks during the treatment period, and at Week 10 (two weeks post-treatment).

### 2.5. Blood Collection and Evaluation

#### 2.5.1. Complete Blood Count and Blood Chemistry

Blood samples were collected from the cephalic and lateral saphenous veins using EDTA tubes for hematology and heparinized tubes for serum biochemistry. Samples were promptly transported to the Small Animal Teaching Hospital, Chulalongkorn University, for analysis. A complete blood count was performed using an automated veterinary hematology analyzer (BC-5000 Vet; Mindray Animal Care, Shenzhen, China) to assess parameters including the red blood cell count (RBC), hematocrit (HCT), and white blood cell count (WBC). Additional hematological indices, such as the hemoglobin concentration (HGB), mean corpuscular volume (MCV), mean corpuscular hemoglobin (MHC), mean corpuscular hemoglobin concentration (MCHC), platelet count, and absolute counts of neutrophils, lymphocytes, monocytes, and eosinophils, were also evaluated. Serum biochemical analysis was conducted using the BC-800 M analyzer (Mindray Animal Care), measuring liver enzymes (alanine aminotransferase and alkaline phosphatase), renal function markers (creatinine and blood urea nitrogen), serum albumin, and total protein concentrations.

#### 2.5.2. Testosterone Levels

For hormone analysis, blood samples were collected twice daily at 7:00 a.m. and 7:00 p.m. during Experiment 1 and weekly between 9:00 and 10:00 a.m. during Experiment 2 using plain tubes (Vacuette^®^; Greiner Bio-One, Chon Buri, Thailand). After clotting, samples were centrifuged at 1000× *g* for 15 min at 4 °C. The resulting sera were harvested and stored at −80 °C until testosterone analysis. Testosterone concentrations were determined using a canine-specific enzyme-linked immunosorbent assay (MBS700523; MyBioSource, San Diego, CA, USA), based on a competitive enzyme immunoassay format. The assay had a detection range of 0.1 to 20 ng/mL and a sensitivity of <0.05 ng/mL, with no significant cross-reactivity or interference from testosterone analogs. Intra-assay and inter-assay coefficients of variation were both below 15%.

### 2.6. Semen Collection and Evaluation

Semen was collected using a digital manipulation technique into 15 mL non-spermicidal, transparent conical plastic tubes with caps (Thermo Scientific™, Waltham, MA, USA). Prior to the pre-treatment phase, an initial semen collection was performed and discarded to eliminate aged sperm residing in the caudal epididymis and vas deferens. This first ejaculate was not used in the analysis. Only samples obtained from subsequent, more consistent collections were included in the pre-, during-, and post-treatment evaluations. Each collection was completed within a fixed 3 min ejaculation window to standardize the sampling time. In Experiment 1 (short-term phase), semen was collected three times—on Days 2, 4, and 9 of the LST treatment period—corresponding to pre-, during-, and post-supplementation time points. In Experiment 2 (long-term phase), semen samples were collected weekly from Week 2 to Week 10. The detailed sampling schedule is illustrated in Figure 1. All procedures—including semen collection and quality assessments—were carried out by the same individual to ensure consistency and minimize variability. Semen samples were evaluated at three time points: immediately after collection, after equilibration, and following frozen–thawed recovery.

#### 2.6.1. Sperm Concentration and Total Number of Sperm per Ejaculate

The sperm concentration was determined using a Neubauer hemocytometer. Semen samples were fixed in a formal saline solution at a 1:20 dilution ratio. Spermatozoa were counted under a phase-contrast microscope at 100× magnification, using 5 of the 25 central squares on the hemocytometer grid. The results were expressed as ×10^6^ spermatozoa per mL. The total number of sperm per ejaculate was calculated by multiplying the sperm concentration by the corresponding semen volume.

#### 2.6.2. Sperm Progressive Motility (%)

An 8 µL aliquot of semen was placed on a pre-warmed microscope slide (37 °C), covered with a coverslip, and examined under a light microscope at 100× magnification. Progressive motility was assessed by observing five randomly selected fields per sample, with a total of 200 sperm cells evaluated to ensure consistency. The percentage of progressively motile spermatozoa—defined as those moving actively in a forward direction—was subjectively estimated for each field and averaged to obtain the final motility percentage.

#### 2.6.3. Sperm Viability (%)

Sperm viability was assessed using a dual fluorescent staining technique with calcein acetoxymethyl ester (Calcein AM), a membrane-permeable dye, and ethidium homodimer-1 (EthD-1), a membrane-impermeable nuclear stain. This method distinguishes viable from non-viable sperm based on plasma membrane integrity. In brief, 10 µL of sperm suspension was mixed with 10 µL of a staining solution containing 2 µM Calcein AM and 4 µM EthD-1 in phosphate-buffered saline (PBS). The mixture was incubated at 37 °C in the dark for 15 min. Following incubation, 5 µL of the stained suspension was mounted on a clean glass slide, covered with a coverslip, and immediately examined under a fluorescence microscope equipped with appropriate filters (excitation/emission: 495/515 nm for Calcein AM and 528/617 nm for EthD-1) at 400× magnification. Viable sperm with intact plasma membranes fluoresced green due to intracellular esterase-mediated conversion of Calcein AM, whereas non-viable sperm with compromised membranes exhibited red nuclear fluorescence from EthD-1. A minimum of 200 sperm per sample was evaluated to calculate the percentages of viable and non-viable cells.

#### 2.6.4. Normal Head Morphology (%)

Sperm head morphology was assessed using William’s staining method and reported as the percentage of spermatozoa with normal head morphology. An 8 µL semen sample was smeared onto a clean glass slide, air-dried, and stained accordingly. In total, 100 spermatozoa per sample were evaluated under a light microscope at 1000× magnification using an oil-immersion objective. Sperm were classified as morphologically normal based on criteria, including a smooth, oval-shaped head with a uniform acrosomal region and an intact post-acrosomal cap.

#### 2.6.5. Normal Tail Morphology (%)

Tail morphology of spermatozoa was assessed by fixing an 8 µL semen sample with formal saline solution. The sample was then mounted on a glass slide, covered with a coverslip, and examined under a phase-contrast microscope. Two hundred spermatozoa were evaluated per sample. Sperm were classified as having normal tail morphology if they exhibited a single, uncoiled, and unbroken tail with no cytoplasmic droplets. Spermatozoa displaying coiled, bent, broken, or multiple tails were classified as having abnormal tail morphology.

#### 2.6.6. DNA Integrity (%)

DNA fragmentation was assessed using the terminal deoxynucleotidyl transferase dUTP nick end labeling (TUNEL) assay. Sperm suspensions were fixed in 4% paraformaldehyde for at least 30 min. Following fixation, the sperm were permeabilized with 0.1% (*v*/*v*) Triton X-100 in PBS for 5 min. DNA fragmentation was then detected using the In Situ Cell Death Detection Kit (Roche, Mannheim, Germany), according to the manufacturer’s protocol. Briefly, sperm samples were incubated with the TUNEL reaction mixture (TdT enzyme to label solution ratio of 1:10, *v*/*v*) for 1 h at 37 °C in a humidified chamber. After TUNEL, the nuclei were counterstained with 4 µM ethidium homodimer-1 (EthD-1). Sperm exhibiting bright green fluorescence were classified as TUNEL-positive, indicating DNA fragmentation or apoptosis, whereas those without green fluorescence were considered TUNEL-negative. A positive control for DNA strand breaks was prepared by treating sperm with 1 mg/mL DNase I overnight prior to TUNEL staining.

#### 2.6.7. Acrosome Integrity (%)

Acrosomal integrity was assessed using fluorescein isothiocyanate-labeled peanut agglutinin (FITC-PNA; *Arachis hypogaea*) staining. A 10 µL aliquot of sperm suspension was mixed with 10 µL of EthD-1 and incubated at 37 °C for 15 min. Following incubation, 5 µL of the mixture was smeared onto a clean glass slide and air-dried. The slides were then fixed in 95% ethanol for 30 s and allowed to air-dry again. Acrosomal staining was performed by applying 50 µL of FITC-PNA solution (10 µg/mL) to each slide, followed by incubation in a dark, humidified chamber at 4 °C for 30 min. Slides were subsequently rinsed with cold phosphate-buffered saline (PBS) and air-dried. Sperm cells were examined under a fluorescence microscope at 1000× magnification

### 2.7. Sperm Cryopreservation and Thawing

The cryopreservation protocol used in this study was slightly modified based on the method by Rota et al. [22] for canine sperm. In the modified extender, glucose was replaced by fructose at an equivalent concentration. Previous studies have indicated that canine sperm metabolize fructose more efficiently than glucose [23]. Extender I contained Tris, citric acid, fructose, glycerol, egg yolk, sodium benzylpenicillin, and streptomycin sulfate dissolved in distilled water. Extender II had the same composition as Extender I, but was modified by adding 1% Equex STM paste and 7% glycerol, resulting in a final glycerol concentration of 5% after mixing sperm with Extenders I and II at a 1:1 ratio. A Tris–fructose–citrate solution, excluding egg yolk and glycerol, was used to mix with thawed sperm in order to mitigate the effects of glycerol and to reduce the osmolarity of the suspension medium. The extended semen was first mixed with Extender I and equilibrated at 4 °C for 1 h. Subsequently, Extender II was added, and the samples were loaded into 0.5 mL straws. To freeze the sperm, the semen-filled straws were layered horizontally to the level of liquid nitrogen, approximately 4 cm above the liquid nitrogen surface, for 10 min. Subsequently, the straws were plunged directly into the liquid nitrogen for storage [22].

For thawing, straws were immersed in a 37 °C water bath for 30 s. Post-thaw assessments were performed at two time points—15 min and 4 h after thawing—to evaluate sperm motility, viability (using calcein–acetoxymethyl ester/ethidium homodimer staining), and morphological integrity under light and fluorescence microscopy. These evaluations provided insights into both immediate and sustained sperm survival following cryopreservation.

### 2.8. Statistical Analysis

Statistical analyses were conducted using SPSS (version 29.0.0.0), and all graphical representations were created with GraphPad Prism (version 9.5.1). Continuous data were assessed for normality and homogeneity of variance, with the results reported as mean ± standard deviation. A multivariable linear regression analysis was employed to evaluate differences across all factors between the pre-treatment, LST treatment, and post-treatment periods. In all cases, differences were considered statistically significant when *p* < 0.05.

## 3. Results

### 3.1. Experiment 1 (Short-Term Administration)

#### 3.1.1. Hematological, Biochemical, and Hormonal Profiles

No clinically relevant alterations were observed in hematological, biochemical, or hormonal parameters following short-term oral administration of LST. Key hematological indices—including RBC, HCT, and WBC—remained within reference ranges throughout the treatment period, with no statistically significant differences from baseline values (*p* > 0.05). Other red cell indices, such as HGB, MCV, MCH, MCHC, and platelet count, also showed no notable changes (*p* > 0.05) (Table 1). Similarly, serum biochemical profiles—including ALT, ALP, creatinine, BUN, albumin, and total protein—remained stable, with no significant differences observed before and after treatment (*p* > 0.05) (Table 1). Serum testosterone concentrations remained within the normal range for adult male dogs as previously reported [24] and demonstrated no statistically significant variation following supplementation (*p* > 0.05) (Table 2).

#### 3.1.2. Semen Characteristics

No statistically significant changes were observed in semen volume, sperm motility, viability, total sperm count per ejaculate, or % head and tail abnormalities following short-term oral administration of LST (*p* > 0.05). All parameters remained within normal biological ranges throughout the pre-treatment, treatment, and post-treatment phases, indicating no adverse effects on semen quality (Table 3).

### 3.2. Experiment 2 (Long-Term Administration)

#### 3.2.1. Hematological, Biochemical, and Hormonal Profiles

Similar to the short-term LST supplementation, hematological values and blood chemistry parameters under the long-term protocol remained within normal ranges (see Table 1). Serum testosterone levels were measured weekly during the LST supplementation period, and no statistically significant differences were observed compared to baseline values (*p* > 0.05) (Table 4).

#### 3.2.2. Prostate and Testicular Morphometry

To evaluate the effects of LST on male reproductive organs, prostatic and testicular volumes were assessed before, during, and after treatment. Prostatic volume showed a slight, progressive increase across the study period; however, the differences were not statistically significant (*p* > 0.05). Similarly, no significant changes were observed in the volumes of the left and right testes throughout the treatment phases (Table 5).

#### 3.2.3. Semen Characteristics

Semen characteristics were evaluated before, during, and after supplementation. Ejaculate volume showed a significant increase during treatment, followed by a moderate reduction in the post-treatment phase (*p* < 0.001). In contrast, other parameters—including sperm motility, viability, total sperm count per ejaculate, and morphology of the head and tail—did not show statistically significant changes. Additionally, acrosome integrity (%) and DNA integrity (%) remained unaffected throughout the study (*p* > 0.05) (Table 6).

#### 3.2.4. Sperm Quality Following Freezing and Thawing

For cryopreservation, no statistically significant differences were observed in post-equilibration motility across the pre-treatment, treatment, and post-treatment groups (*p* > 0.05). However, a significant improvement in sperm motility was observed at 15 min after thawing in the post-treatment group (*p* < 0.01). Despite this improvement in motility, sperm viability did not differ significantly among the groups (*p* > 0.05), although the post-treatment group showed the numerically highest viability. At 4 h post-thawing, sperm motility in the post-treatment group was significantly higher compared to the pre-treatment and treatment groups (*p* < 0.05). Likewise, sperm viability at 4 h post-thawing was significantly improved in the post-treatment phase compared to the earlier phases (Table 7).

## 4. Discussion

This study explores the effects of short- and long-term supplementation of LST on hematological values, semen characteristics, testicular and prostatic parameters, and the cryopreservation potential of canine sperm. Neither short- nor long-term administration of LST resulted in significant changes in hematological parameters, suggesting that LST did not adversely affect general health status, even after 60 days of continuous supplementation. This observation aligns with studies in other species; for example, research in male rats found no toxicological effects or adverse health impacts associated with LST supplementation [25]. Such cross-species consistency supports the hypothesis that LST is a safe supplement, even for extended durations, reinforcing its potential as a non-toxic therapeutic option in various applications.

In this study, testosterone levels were not significantly different between the pre-treatment and LST treatment groups; the values remained within the established physiological range for intact male dogs (0.4–6.0 ng/mL) [24]. The lack of change in testosterone levels is likely due to the operation of negative feedback loops within the hypothalamic–pituitary–gonadal axis, which suppress testosterone secretion in response to elevated androgen levels [26]. These results are consistent with findings in buffalo calves [20], roosters [16], and rats [25]. In contrast, previous studies on rams have shown that LST exhibits phytoandrogenic properties, resulting in a significant increase in plasma testosterone [18]. These differences suggest that the androgenic effects of LST may vary across species and could be influenced by dose–response dynamics. In dogs [27], swamp buffalo [17], and rams [18], doses as high as 50 mg/kg have been used. However, due to the probable risks associated with long-term use in dogs—and considering the observed effectiveness of LST in improving sperm quality after cryopreservation—a dose of 10 mg/kg was selected. Furthermore, based on our preliminary short-term study in dogs comparing oral doses of 10 mg/kg and 20 mg/kg, no statistically significant differences in semen volume or sperm quality were observed between the two groups (see Figure S1). To minimize risk, the 10 mg/kg dose was adopted in the present study, not only for its effectiveness but also to reduce potential adverse effects and to prioritize animal welfare.

LST treatment was associated with an increase in prostate gland volume, which may explain the elevated ejaculate volume observed, as prostatic fluid constitutes the majority of canine semen and greatly influences its quality. However, the exact mechanisms underlying this effect remain unclear. Although LST contains phytochemicals with potential androgen-like or reproductive-modulating properties, no significant increase in serum testosterone was detected in treated dogs [28]. The mild enlargement of the prostate gland observed may therefore reflect local paracrine effects or the subtle bioactivity of steroidal constituents, rather than systemic androgen stimulation. In contrast, no significant increase in testicular volume was observed in this study, despite the potential androgenic effects of LST. This finding differs from previous studies in rats, where similar treatments led to increased testicular size, suggesting possible species-specific responses to androgenic stimulation [25]. Fresh semen quality analysis revealed an increase in semen volume following LST administration, consistent with findings reported in boars [15] and roosters [16].

This study is the first to report that LST enhances the cryopreservation ability of dog sperm, notably improving post-thaw motility and viability after long-term treatment. LST may exert protective effects during cryopreservation through mechanisms involving membrane stabilization and reduction in oxidative stress. Previous studies have demonstrated the antioxidative properties of LST, particularly due to its rich polyphenol content, which contributes to its ability to reduce oxidative damage. The phenolic compounds in LST, known for their antioxidant properties, are beneficial in maintaining sperm membrane integrity [19]. Elevated levels of reactive oxygen species are known to impair critical aspects of sperm function, including motility, hyperactivation, capacitation, the acrosome reaction, and DNA integrity (%) [29,30]. Although no improvements were observed in fresh semen quality parameters, the observed enhancement in post-thaw viability and motility suggests the potential efficacy of LST in supporting sperm preservation during cryopreservation. This protective effect is particularly valuable because cryopreservation commonly leads to structural damage and reduced viability due to increased oxidative stress. These findings highlight LST’s potential as a valuable supplement for enhancing sperm resilience to cryogenic stress. To further elucidate the underlying mechanisms of this protective effect, future studies should incorporate direct assessments of oxidative stress markers and sperm structural integrity. ROS-specific assays—such as BODIPY 581/591, TBARS, CM-H_2_DCFDA, or CellROX Green—could provide insights into the degree of oxidative stress and the antioxidant efficacy of LST at the cellular level. In addition, evaluating sperm acrosome integrity (%) and DNA fragmentation would offer a more comprehensive understanding of how LST influences cryotolerance, particularly in terms of preserving sperm morphology and genetic stability during the freezing and thawing processes.

The limitations of this study include the small sample size, which consisted exclusively of healthy, medium-sized dogs aged 2 to 3 years. Therefore, the findings may not be representative of older dogs or those with infertility issues. In addition, the total number of spermatozoa per ejaculate observed in this study appeared to be lower than values reported in other studies on medium-sized dogs [3,31]. This may be partly attributable to the fixed 3 min ejaculation window applied during semen collection, which could have limited the total sperm yield. Furthermore, the limited pharmacokinetic and toxicological data on LST in dogs—including absorption, distribution, metabolism, excretion, and long-term safety—represent a critical gap. Future studies are needed to provide a more comprehensive understanding of the efficacy, safety, and mechanisms of action of LST in canine reproductive management.

## 5. Conclusions

This study shows that LST supplementation can improve the quality of frozen–thawed canine sperm, particularly by enhancing post-thaw motility and viability. Over 60 days of treatment, LST did not significantly affect hematological parameters, blood chemistry, testosterone levels, or reproductive structures, with no apparent adverse effects under short- and long-term clinical observation. These results indicate a possible protective effect of LST on sperm during cryopreservation.

## Figures and Tables

**Table 1:** Hematological and blood chemistry profiles of dogs following short-term LST supplementation (7 days) and long-term LST supplementation (60 days).

Hematological Parameters	Reference Interval	Short-Term			Long-Term		
		Pre-Treatment	LST Treatment	Post-Treatment	Pre-Treatment	LST Treatment	Post-Treatment
RBC (×10^6^/µL)	4.95–7.87	7.85 ± 0.63	7.66 ± 0.45	7.63 ± 0.5	6.94 ± 0.84	6.20 ± 0.53	6.80 ± 0.71
HGB (g/dL)	11.9–18.9	16.2 ± 0.72	16.13 ± 0.45	16.2 ± 0.75	14.80 ± 1.53	12.8 ± 0.78	14.20 ± 1.13
HCT (%)	35–57	49.00 ± 2.17	45.27 ± 1.79	45.13 ± 2.17	41.55 ± 3.65	39.07 ± 1.57	44.15 ± 0.50
MCV (fl)	66–7	62.57 ± 2.85	59.13 ± 1.36	59.17 ± 2.14	60.08 ± 2.14	63.20 ± 3.03	65.30 ± 7.50
MCH (pg)	21.0–26.2	20.63 ± 0.75	21.07 ± 0.72	21.23 ± 0.93	21.38 ± 0.92	20.70 ± 0.60	20.85 ± 0.50
MCHC (g/L)	32.0–36.3	33.00 ± 0.5	35.63 ± 0.76	35.87 ± 0.29	35.60 ± 0.94	32.80 ± 0.70	32.10 ± 2.97
WBC (×10^3^/µL)	5.1–14.1	12.53 ± 0.56	10.93 ± 1.15	13.14 ± 1.71	11.57 ± 1.77	10.15 ± 2.56	12.90 ± 1.50
Absolute neutrophils (×10^3^/µL)	2.9–12.0	7.17 ± 0.49	6.09 ± 1.07	6.44 ± 1.33	7.16 ± 1.72	6.17 ± 1.51	7.66 ± 0.60
Absolute lymphocytes (×10^3^/µL)	0.4–2.9	2.05 ± 0.51	2.93 ± 1.21	2.32 ± 1.48	2.30 ± 0.65	2.6 ± 0.69	3.81 ± 1.90
Absolute monocytes (×10^3^/µL)	0.1–1.4	0.377 ± 0.59	0.39 ± 0.72	0.393 ± 0.68	1.01 ± 0.2	0.60 ± 0.17	0.87 ± 0.33
Absolute eosinophils (×10^3^/µL)	0.0–1.3	0.39 ± 0.17	0.57 ± 1.78	0.83 ± 0.77	1.09 ± 0.71	0.72 ± 1.87	0.81 ± 0.22
Platelets (×10^3^/µL)	160–525	233.67 ± 40.43	275.00 ± 22.11	252.00 ± 38.43	231.00 ± 32.47	252.33 ± 68.30	269.58 ± 51.66
ALT (U/I)	10–109	43.00 ± 4.58	37.67 ± 3.21	36.67 ± 1.15	54.33 ± 20.30	44.92 ± 8.7.8	47.45 ± 9.64
ALP (U/I)	1–114	24.00 ± 14.18	16.67 ± 4.73	22.00 ± 6.56	44.50 ^±^ 8.27	41.67 ± 2.89	48.50 ± 19.10
Creatinine (mg%)	0.5–17	1.10 ± 0.10	1.10 ± 0.00	1.13 ± 0.12	1.06 ± 0.10	0.95 ± 0.18	1.09 ± 0.33
BUN (mg%)	8–28	19.60 ± 1.91	22.80 ± 7.64	15.97 ± 4.91	20.37 ± 6.06	18.32 ± 4.29	19.00 ± 4.42
Albumin (g%)	2.3–3.1	2.47 ± 0.15	2.50 ± 0.17	2.57 ± 0.25	2.71 ± 0.21	2.65 ± 0.20	2.59 ± 0.26
Total protein (g%)	6.0–5.7	6.30 ± 0.17	6.40 ± 0.20	6.20 ± 0.36	6.58 ± 0.43	6.57 ± 0.60	6.35 ± 0.07

Values are presented as mean ± SD. No significant differences were observed among time points (*p* > 0.05).

**Table 2:** Serum testosterone levels during short-term LST supplementation.

Parameter	Pre-Treatment	LST Treatment			
	0 h	12 h	24 h	36 h	48 h
Testosterone level (ng/mL)	8.15 ± 3.91	7.97± 1.33	7.60 ± 3.46	3.18 ± 0.87	9.37 ± 1.48

Values are presented as mean ± SD. No significant differences were observed among time points (*p* > 0.05).

**Table 3:** Comparison of semen quality parameters across pre-treatment, LST treatment, and post-treatment phases (short-term administration).

Parameter	Pre-Treatment	LST Treatment	Post-Treatment
Volume (mL)	3.48 ± 0.50	4.17 ± 1.26	4.43 ± 4.39
Motility (%)	88.33 ± 2.89	90.00 ± 0.00	86.67 ± 5.77
Viability (%)	96.97 ± 1.11	89.26 ± 5.79	89.40 ± 8.79
Total sperm/ejaculate (×10^6^)	445.48 ± 307.69	312.57 ± 85.31	296.00 ± 52.85
Normal head morphology (%)	97.97 ± 0.59	98.50 ± 0.40	97.60 ± 0.44
Normal tail morphology (%)	85.49 ± 3.61	88.38 ± 1.10	88.58 ± 4.08

Values are presented as mean ± SD. No significant differences were observed among time points (*p* > 0.05).

**Table 4:** Serum testosterone levels during long-term LST supplementation.

Parameter	Pre-Treatment	LST Treatment				
	Week 0	Week 1	Week 2	Week 3	Week 4	Week 5
Testosterone level (ng/mL)	4.38 ± 0.60	3.09 ± 1.17	3.64 ± 1.34	5.70 ± 3.57	3.89 ± 2.78	4.11 ± 3.02

Values are presented as mean ± SD. No significant differences were observed among time points (*p* > 0.05).

**Table 5:** Prostatic and testicular volumes in dogs at pre-, during-, and post-LST treatment phases during long-term LST supplementation.

Reproductive Organ Volume (cm^3^)	Pre-Treatment	LST Treatment	Post-Treatment
Prostatic volume	12.56 ± 2.44	14.65 ± 4.52	15.28 ± 3.57
Left testis volume	18.59 ± 7.43	18.87 ± 7.32	20.85 ± 9.33
Right testis volume	19.61 ± 5.24	20.95 ± 4.96	23.84 ± 6.68

Values are presented as mean ± SD. No significant differences were observed among time points (*p* > 0.05).

**Table 6:** Semen quality comparison across pre-treatment, LST treatment, and post-treatment phases (long-term administration).

Parameter	Pre-Treatment	LST Treatment	Post-Treatment
Volume/ejaculate (mL)	4.10 ^a^ ± 2.15	13.25 ^b^ ± 0.35	10.60 ^b^ ± 3.77
Motility (%)	82.50 ^a^ ± 13.89	90.00 ^a^ ± 0.00	90.00 ^a^ ± 0.00
Viability (%)	90.69 ^a^ ± 7.94	93.35 ^a^ ± 2.33	93.64 ^a^ ± 1.76
Total sperm/ejaculate (×10^6^)	394.92 ^a^ ± 180.34	289.70 ^a^ ± 3.96	433.68 ^a^ ± 142.80
Normal head morphology (%)	96.08 ^a^ ± 1.60	97.15 ^a^ ± 0.64	95.00 ^a^ ± 2.82
Normal tail morphology (%)	89.83 ^a^ ± 5.74	90.40 ^a^ ± 3.82	94.14 ^a^ ± 2.91
Acrosome integrity (%)	94.60 ^a^ ± 2.05	97.00 ^a^ ± 1.63	94.26 ^a^ ± 1.50
DNA integrity (%)	96.90 ^a^ ± 1.22	95.00 ^a^ ± 0.28	96.00 ^a^ ± 1.90

Superscript letters indicate statistically significant differences within the same row. Values with different superscript letters (^a, b^) are significantly different between treatments (*p* < 0.05).

**Table 7:** The sperm quality after cryopreservation comparison across pre-treatment, LST treatment, and post-treatment phases (Long-term administration).

Parameter	Pre-Treatment	LST Treatment	Post-Treatment
Post equilibration motility (%)	70.00 ^a^ ± 17.72	80.00 ^a^ ± 0.00	84.00 ^a^ ± 5.48
Motility 15 min post-thawing (%)	51.25 ^a^ ± 6.41	50.00 ^a^ ± 0.00	66.00 ^b^ ± 5.5
Viability 15 min post-thawing (%)	74.25 ^a^ ± 9.23	63.00 ^a^ ± 4.95	77.70 ^a^ ± 10.50
Motility 4 h post-thawing (%)	33.75 ^a^ ± 7.44	35.00 ^a^ ± 7.07	46.00 ^b^ ± 5.50
Viability 4 h post-thawing (%)	48.13 ^a^ ± 4.57	45.65 ^a^ ± 2.05	61.90 ^b^ ± 0.94

Superscript letters indicate statistically significant differences within the same row. Values with different superscript letters (**^a^**^,^ **^b^**) are significantly different between treatments (*p* < 0.05).

## Data Availability

The original contributions presented in this study are included in the Supplementary Materials. Further inquiries can be directed to the corresponding author.

## Supplementary Materials

The following supporting information can be downloaded at: https://www.mdpi.com/article/10.3390/ani15162379/s1, Figure S1: Effects of short-term LST administration (10 mg/kg and 20 mg/kg) on semen concentration and quality in dogs. No statistically significant differences were observed between the two groups.

## Abbreviations

The following abbreviations are used in this manuscript:

ARRIVE	Animal Research: Reporting of In Vivo Experiments
DPPH	2,2-diphenyl-1-picrylhydrazyl
EDTA	Ethylenediaminetetraacetic Acid
IACUC	Institutional Animal Care and Use Committee
LST	*Lasia spinosa* Thwaites
RBC	Red blood cell count
WBC	White blood cell count
SPSS	Statistical Package for the Social Sciences
ng/mL	Nanograms per milliliter
µL	Microliter
mL	Milliliter
g/dL	Grams per deciliter
U/L	Units per liter
mg/dL	Milligrams per deciliter
cm^3^	Cubic centimeters
°C	Degrees Celsius
