# Supplementary material for: Oral Supplementation of Lasia spinosa Thwaites Improves Sperm Cryotolerance Without Markedly Affecting Hematological, Biochemical, Seminal, or Testicular Profiles in Dogs
Source: Animals (Basel). 2025 Aug 13;15(16):2379. doi: 10.3390/ani15162379 (PMC12382832; doi:10.3390/ani15162379)
Supplement: Supplementary file 1 [file animals-15-02379-s001.zip › animals-3733777-supplementary.pdf]

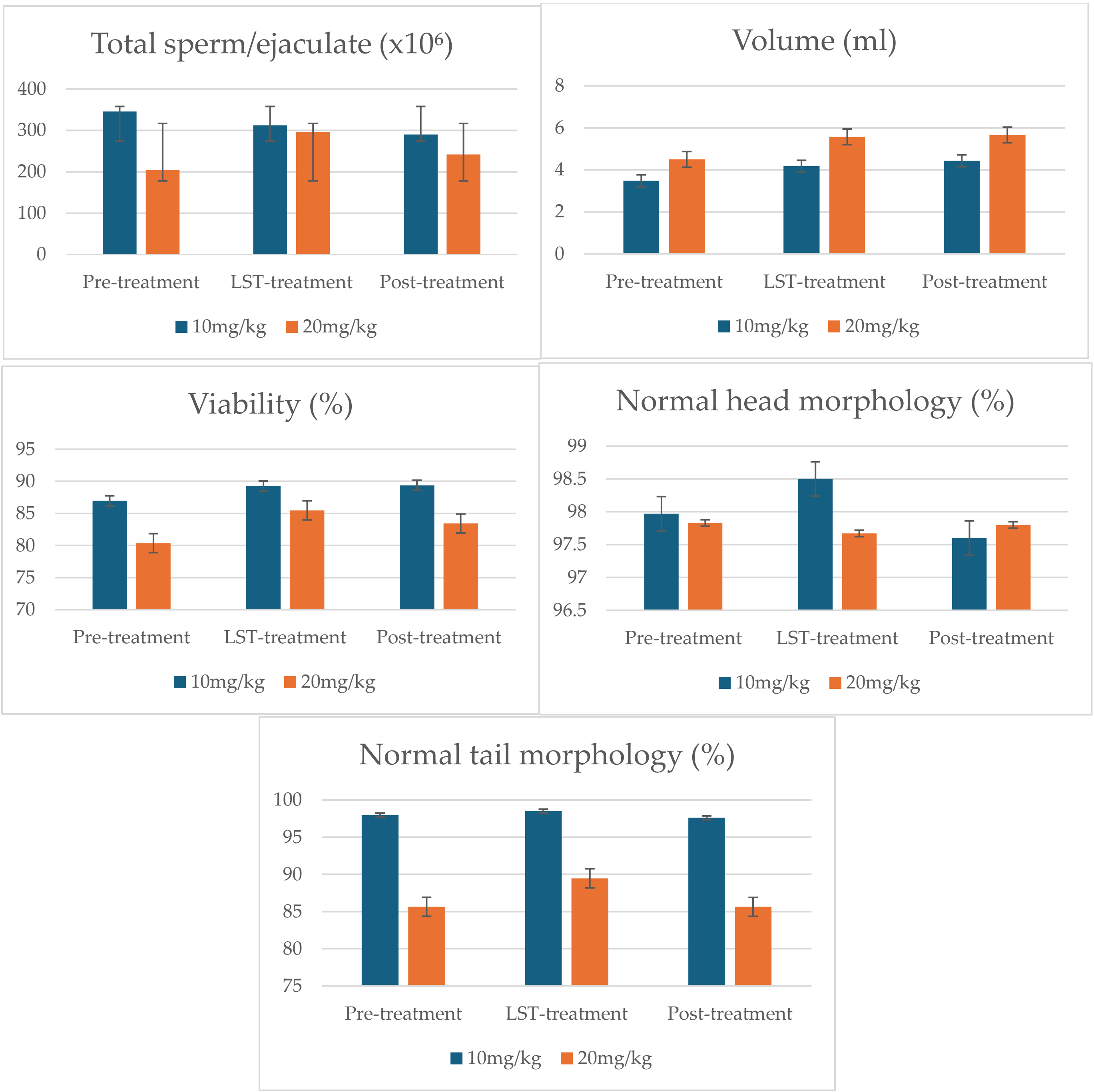

Figure S1. Effects of short-term LST administration (10 mg/kg and 20 mg/kg) on semen concentration and quality in dogs. No statistically significant differences were observed between the two groups.
